# Supplementary material for: In-vitro diagnostic point-of-care tests in paediatric ambulatory care: A systematic review and meta-analysis
Source: PLoS One. 2020 Jul 6;15(7):e0235605. doi: 10.1371/journal.pone.0235605 (PMC7337322; doi:10.1371/journal.pone.0235605)
Supplement: S1 File — (DOCX) [file pone.0235605.s001.docx]

**Supplementary Appendix**

Table of Contents

[S1. Summary of point-of-care tests and the principle method used 2](#_Toc36745674)

[S2. Description of the role of POCT in clinical pathway and associated educational component in addition to POCT training 7](#_Toc36745675)

[S3. Further information on patient outcomes in malarial studies 14](#_Toc36745676)

[S4. Timeliness of appropriate antimalarial treatment 15](#_Toc36745677)

[S5. Safety and antimalarial treatment 16](#_Toc36745678)

[S6. Additional information about point-of-care testing C-reactive protein in non-specific acute fever illness 20](#_Toc36745679)

[S7. POCT-Hba1c and Diabetes Mellitus 21](#_Toc36745680)

[S8. Search strategy example of electronic database (Medline) 22](#_Toc36745681)

## **S1. Summary of point-of-care tests and the principle method used**

All point-of-care malaria devices used an immunochromatographic assay using capillary whole blood to either detect histidine-rich protein (HRP-2) produced by *Plasmodium falciparum*, or parasite lactate dehydrogenase (pLDH, panmalarial antigen). Two studies used 2 or more types of point-of-care malaria devices (Mukanga, Tiono et al. 2012, Baiden, Bruce et al. 2016) and two studies did not report the type of POCT used.(Mbonye, Magnussen et al. 2015, Chandler, Webb et al. 2017) The two HIV studies used the same nucleic acid-based HIV POCT test.(Jani, Meggi et al. 2018, Mwenda, Fong et al. 2018) All studies using CRP-POCT employed a quantitative immunochemical assay for C-reactive protein.(Diederichsen, Skamling et al. 2000, Cohen, Lecuyer et al. 2008, Nijman, Moll et al. 2015, Do, Ta et al. 2016, Van den Bruel, Jones et al. 2016, Verbakel, Lemiengre et al. 2016, Rebnord, Sandvik et al. 2017, Lemiengre, Verbakel et al. 2018) Four studies used a rapid antigen test for Strep A (Ayanruah 2009; Malecki 2017; Maltezou 2008; Meier 1990). One study used a panel test, a direct immunofluorescence assay targeting adenovirus, respiratory syncytial virus, parainfluenza and influenza (Doan 2009), and one study used POCT Hba1c.(Agus, Alexander et al. 2010)

**Table S1. Description of point-of-care tests and the principle method used**

| Study | Point-of-care test | Manufacturer | Method principle | Sample type |
| --- | --- | --- | --- | --- |
| Malaria (Pf) |  |  |  |  |
| Ansah et al. 2010 | OptiMAL-IT | BIO-RAD; California, USA | OptiMAL-IT is an immuno-chromatographic test, using monoclonal antibodies against the metabolic enzyme pLDH (parasite lactate dehydrogenase) of Plasmodium spp. | Capillary Whole blood |
| Ansah et al. 2015 | CareStart Malaria HRP2 | Access Bio; New Jersey, USA | CareStart™RDT is an immunochromatographic test coated with monoclonal antibodies in two separate bands, one recognizing the specific histidine-rich protein-2 (HRP-2) associated with the presence of Plasmodium falciparum and the other detecting presence of pan malaria-specific antigen (pLDH) of all malarial parasites species. | Capillary Whole blood |
| Baiden et al. 2016 | 1. CareStart Malaria and 2. First Response | 1. Access Bio; New Jersey, USA; 2. Premier Medical Corporation; Daman, India | See above.  First Response principle of immunochromatography in which nitrocellulose membrane is pre-coated with one monoclonal antibody (test line P.f.) specific to Histidine Rich Protein 2 (HRP2) of the Plasmodium falciparum. | a. Capillary Whole blood  b. Whole blood |
| Chandler et al. 2017 | Not reported | Not applicable | Not applicable | Not applicable |
| Hopkins et al. 2017 | SD Bioline Pf Standard Diagnostics | SD Diagnostics; Korea | The SD BIOLINE Malaria Ag P.f test is a rapid, qualitative test for the detection of histidine-rich protein II (HRP-II) antigen of Malaria Plasmodium falciparum in human whole blood. | Capillary Whole blood |
| Lal et al. 2016 | First Response® Malaria HRP2 | Premier Medical Corporation; Daman, India | First Response principle of immunochromatography in which nitrocellulose membrane is pre-coated with one monoclonal antibody (test line Pf.) specific to Histidine Rich Protein 2 (HRP2) of the Plasmodium falciparum. | Whole blood |
| Mbonye et al. 2015 | Not reported | Not applicable | Not applicable | Not applicable |
| Msellum et al. 2009 | Paracheck Pf | Orchid Biomedical Systems; India | Histidine-rich protein (HRP) 2 based test | Capillary Whole Blood |
| Mubi et al. 2011 | Paracheck Pf | Orchid Biomedical Systems; India | Histidine-rich protein (HRP) 2 based test | Capillary Whole Blood |
| Mukanga et al. 2012 | a. First Sign Malaria Pf Card Test;  b. Paracheck Pf;  c. ICT Malaria Pf) | a. Unimed International Inc, South San Francisco, USA  b. Orchid Biomedical Systems; India  c. ICT Diagnostics; New South Wales, Australia | a. FirstSign™ Malaria Pf rapid diagnostic test detects the P. falciparum-specific histidine-rich protein 2 (HRP-2)  b. Histidine-rich protein (HRP) 2 based test  c. Immunochromatographic assay detecting Plasmodium falciparum-specific histidine-rich protein 2 antigen (HRP2) and a panmalarial antigen. | a. Capillary Whole Blood  b. Capillary Whole Blood  c. Capillary Whole blood/venous |
| Ndyomugyenyi et al. 2016 | First Response Malaria HRP2 | Premier Medical Corporation; Daman, India | First Response principle of immunochromatography in which nitrocellulose membrane is pre-coated with one monoclonal antibody (test line Pf.) specific to Histidine Rich Protein 2 (HRP2) of the Plasmodium falciparum. | Whole blood |
| Sayang et al. 2009 | Diaspot Malaria RDT cassette | Acumen Diagnostics Inc; USA | Diaspot is based on the detection of Pf HRP-2. | Capillary Whole Blood |
| Ukwaja et al. 2010 | Paracheck Pf | Orchid Biomedical Systems; India | Histidine-rich protein (HRP) 2 based test | Capillary Whole Blood |
| Yeboah-Antwi et al. 2010 | ICT Malaria Pf | ICT Diagnostics; New South Wales, Australia | Immunochromatographic assay detecting Plasmodium falciparum-specific histidine-rich protein 2 antigen (HRP2) and a panmalarial antigen. | Capillary Whole blood/venous |
| Non-specific fever ‘illness’ |  |  |  |  |
| Althaus et al. 2019 | Nyocard CRP analyser | Alere (now Abbott) | Quantitative immunochemical assay for C-reactive protein | Capillary Whole Blood |
| Cohen et al. 2008 | Nyocard CRP analyser | Alere (now Abbott) | Quantitative immunochemical assay for C-reactive protein | Capillary Whole Blood |
| Lemiengre et al. 2018 | Afinion AS 100 CRP analyser | Alere (now Abbott) | Quantitative immunochemical assay for C-reactive protein | Capillary Whole Blood |
| Nijman et al. 2015 | Afinion AS 100 CRP analyser | Alere (now Abbott) | Quantitative immunochemical assay for C-reactive protein | Capillary Whole Blood |
| Rebnord et al. 2017 | QuikRead Go CRP | Orion Diagnostica | Quantitative immunochemical assay for C-reactive protein | Capillary Whole Blood |
| Van den Bruel et al. 2016 | Afinion AS 100 CRP analyser | Alere (now Abbott) | Quantitative immunochemical assay for C-reactive protein | Capillary Whole Blood |
| Verbakel et al. 2016 | Afinion AS 100 CRP analyser | Alere (now Abbott) | Quantitative immunochemical assay for C-reactive protein | Capillary Whole Blood |
| Acute RTIs |  |  |  |  |
| Diederichsen et al. 2000 | NycoCard CRP  II | Alere (now Abbott) |  | Capillary Whole Blood |
| Do et al. 2016 | Nyocard CRP analyser | Alere (now Abbott) | Quantitative immunochemical assay for C-reactive protein | Capillary Whole Blood |
| Doan et al. 2009 | Panel test for Adenovirus, Influenza A/B, parainfluenza 1/2/3, RSV |  | Direct immunofluorescence assay targeting adenovirus, respiratory syncytial virus, parainfluenza and influenza |  |
| Keitel et al. 2019 | BioNexia CRP plus analyser | bioMérieux | Quantitative immunochemical assay for C-reactive protein | Capillary Whole Blood |
| Schot et al. 2018 | Afinion AS 100 CRP analyser | Alere (now Abbott) | Quantitative immunochemical assay for C-reactive protein | Capillary Whole Blood |
| Acute sore throat |  |  |  |  |
| Bird et al. 2018 | BioNexia Strep A test | bioMérieux, | Qualitative detection of group A Streptococcal antigen (GAS) | Throat swab |
| Ayanruoh et al. 2009 | Rapid streptococcal test | Sacks Biological Farms | Rapid enzyme-linked immunosorbent assay | Throat swab |
| Malecki et al. 2017 | OSOM Strep A test | Sekisui Diagnostics | Chromatographic immunoassay for detection of Group A Streptococcus antigen | Throat swab |
| Maltezou et al. 2008 | BD Link 2 Strep A Rapid antigen test | Becton Dickinson | Chromatographic immunoassay for detection of Group A Streptococcus antigen | Throat swab |
| Meier et al. 1990 | Culturette | Marion Laboratories | Latex agglutination antigen detection method | Throat swab |
| HIV |  |  |  |  |
| Bianchi et al. 2019 | ^1^m-PIMA HIV-1/2 Detect  ^2^Xpert HIV-1 Qual | ^1^Abbott  ^2^Cepheid | Qualitative nucleic acid amplification test for the detection of human immunodeficiency virus (HIV) type 1 groups M/N and O, and type 2 | Capillary or venous blood |
| Jani et al. 2018 | Alere q HIV 1/2 Detect System | Alere | Qualitative nucleic acid amplification test for the detection of human immunodeficiency virus (HIV) type 1 groups M/N and O, and type 2 | Capillary or venous blood |
| Mwenda et al. 2018 | Alere q HIV 1/2 Detect System | Alere | Qualitative nucleic acid amplification test for the detection of human immunodeficiency virus (HIV) type 1 groups M/N and O, and type 2 | Capillary or venous blood |
| Insulin-dependent diabetes mellitus |  |  |  |  |
| Agus et al. 2010 | POCT Hba1C, DCA2000+ Analyser | Bayer Diagnostics Europe, Ireland | Immunoassay method using antibodies which bind to the N-terminal glycated tetrapeptide or hexapeptide group of the HbA1c, forming immunocomplexes which can be detected and measured using a turbidimeter or a nephelometer. | Capillary or venous blood |

## **S2. Description of the role of POCT in clinical pathway and associated educational component in addition to POCT training**

| **Study** | **Role in**  **clinical pathway^a^** | **Description of POC role (where reported)** | **Education component in addition to POCT training** |
| --- | --- | --- | --- |
| **Malaria (Pf)** |  |  |  |
| Ansah et al. 2010 | Replacement | Eligible children meeting inclusion criteria 1:1 randomisation to malaria-POCT or microscopy, after examination | All healthcare professionals in participating centres were given identical training about rapid diagnostic tests, alternative causes of febrile illness, and the Ghana national guidelines, which indicate presumptive treatment for children under 5 years of age . |
| Ansah et al. 2015 | Triage | Shops in communities carried out malarial POCT before dispensing any medication. | All sellers attended a three-day training on malaria, covering topics that included the antimalarial drug policy of Ghana; signs and symptoms of malaria; signs of severe disease and indications for referral; how to take a blood sample and make a blood slide; blood safety and handling of sharps; infection prevention procedures  Sellers in the intervention arm attended an additional day of training, covering how to carry out and interpret rapid diagnostic tests for malaria and further management of clients with a negative rapid diagnostic test result. |
| Baiden et al. 2016 | Add-on | Fieldworkers conducted sampling from eligible children. Results were made available to the attending health worker. | Trained study field-workers on how to take blood from finger prick to perform malaria POCTs, and how to prepare blood smears for microscopy.  Refresher training for healthcare workers in the management of febrile illnesses according to IMCI guidelines, repeated every 4–6 months throughout the period of the study  Clinicians in all the participating health facilities were taken through the correct use and interpretation of RDT. |
| Chandler et al. 2017 | Add-on | Children and their caregivers were approached by study personnel as they are leaving health centres.  A clinical evaluation of the child was performed by a study physician as part of the exit interview. If the child had a temperature of ≥38.0°C or a history of fever in the past 48 hours, a finger-prick blood sample was obtained to perform a malaria POCTs. The results were compared to the results of POCTs performed by health facility staff, where possible. | The intervention package included amongst other things: training health workers in fever case management, including differential diagnosis, and use of malarial POCTs,  No additional training will be provided to control clinics. |
| Hopkins et al. 2017 | Add-on/Replacement ^c^ | Not reported. Febrile patients at randomly selected health facilities were interviewed about care received at the facility. | Two-day Ministry of Health training in intervention settings: performing malarial POCTs, prescribing antimalarials, rationale for malaria guideline change, identifying major alternative causes of febrile illness |
| Lal et al. 2016 | Add-on | Not reported. Assumed that POCT use would follow clinical examination. | All newly recruited community health workers (CHW) received training in the management of febrile children during 3- to 4-day workshops. The training was based on a manual with simplified pictorial treatment algorithms (job aids) to help with malaria diagnosis. The key topics included how to take a basic clinical history and physical examination skills, All CHWs were also trained on how to identify non-severe and severe signs and symptoms, which would require immediate referral.  CHWs in the control arm were trained to make a presumptive diagnosis based on the clinical symptoms of malaria if a child had a fever or history of fever without any other obvious causes of fever and prescribe accordingly; CHWs in the intervention arm were trained to diagnose uncomplicated malaria with a malaria POCT. CHWs were trained not to prescribe ACT to children who were POCT negative, and to refer those who had signs and symptoms of severe or non-severe illnesses. |
| Mbonye et al. 2015 | Add-on | Not reported. Drug shop vendors were required to record symptoms. | Training provided to participatory drug shops in both arms based on a trainer’s manual and accompanying set of pictorial job aids on malaria case management. Vendors in the control arm were trained in presumptive diagnosis of malaria.  The intervention arm received additional one-day training to cover the rationale for diagnostic testing in febrile patients, performing a malaria POCT and interpretation of the test result, and necessary communication skills to explain the rationale for diagnostic testing and treatments given. |
| Msellum et al. 2009 | Add-on | Each health facility alternately used malaria POCTs or usual care alone on a weekly basis. | Pre-study training of nurses in malaria case management according to the Ministry of Health. In addition, and prior to the study start the staff members of all four health facilities received a one-day training on the use of POCTs including performance of the test and interpretation of the result. |
| Mubi et al. 2011 | Add-on | CHWs provided treatment based on symptoms together with an POCT positive result. | CHWs received 1-week specific training on malaria symptoms, performance and interpretation of malaria POCTs, prescription of ACT (artemether-lumefantrine), identification of danger signs according to IMCI and indications for referral. |
| Mukanga et al. 2012 | Add-on | CHWs assessed children with acute febrile illness for malaria using malarial POCTs. Treatment was provided on the basis of the test results. | Training provided to CHW included history-taking, clinical features of uncomplicated malaria, and signs of severe illness requiring referral.  In addition, CHWs in the intervention arm were taught the use of malaria POCTs; infection control measures; how to count respiratory rate; and the use of simple dosing guidelines based on age for antibiotics.  At the health facility level, health personnel were oriented on the treatment strategies in the two arms, and received refresher training on IMCI. |
| Ndyomugyenyi et al. 2016 | Add-on | CHWs assessed children with acute febrile illness for malaria using malarial POCTs. Treatment was provided on the basis of the test result. | Training provided to community health workers in both arms of the trial in recognising signs and symptoms of malaria, how to administer antimalarial treatment and when to refer.  Additional training intervention arm only about in malaria diagnosis using POCT, interpretation, and included practice in communication skills necessary to explain the rationale for diagnostic testing, POCT and treatments given. CHWs were trained to only give antimalarial treatment with ACT after a positive test result. |
| Sayang et al. 2009 | Add-on | Healthcare staff assessed children with acute febrile illness for malaria using malarial POCTs. Treatment was provided on the basis of the test result. | A training session on the use of POCT for malaria took place two weeks before the beginning of the study. |
| Ukwaja et al. 2010 | Add-on | Healthcare staff assessed eligible children and based treatment on malaria POCT | Not reported. |
| Yeboah-Antwi et al. 2010 | Add-on | Intervention CHWs performed malaria POCTss on children with reported fever. | All study CHWs participated in an additional 5-d training workshop using modifications of nationally developed training manuals.  The training emphasized community-based integrated management of febrile children including basic clinical history taking, physical examination skills, treatment algorithms, counselling of caregivers, and recognition of signs of severe illness requiring referral.  Additional training was provided to the intervention group only. This included performing and interpreting POCTs, and how to action both positive and negative results.  After completion of training, instructors assessed the competency of all CHWs and a follow-up assessment at 1 month after initial training.  All study CHWs completed an additional 2-d refresher course 6 months after the initial training. |
| **Non-specific fever ‘illness’** |  |  |  |
| Althaus et al. 2019 | Triage | Patients allocated to two pre-defined CRP-POCT thresholds based on CRP concentrations before medical examination | Yes, for clinicians and patients |
| Cohen et al. 2008 | Replacement | ‘During clinical consultations ‘ | Not reported |
| Lemiengre et al. 2018 | Add-on | Not reported | Brief intervention to elicit parental concern combined with safety net advice |
| Nijman et al. 2015 | Triage | Study protocol to guide trained nurses in performing a POC CRP at triage | Not reported |
| Rebnord et al. 2017 | Add-on | POC CRP test on every third child before the consultation in intervention arm | Not reported |
| Van den Bruel et al. 2016 | Triage | Eligible children 1:1 randomisation to CRP testing, prior to examination | Not reported |
| Verbakel et al. 2016 | Triage | Eligible children 1:1 randomisation to CRP testing, prior to examination | POC training only |
| **Acute RTIs** |  |  |  |
| Diederichsen et al. 2000 | Add-on | POC CRP combined with a clinical assessment | Yes, for clinicians only |
| Do et al. 2016 | Add-on | POC CRP combined with a clinical assessment | Yes. Physicians were trained to use specific CRP cut-offs, based on previous studies and adapted for use in children. |
| Doan et al. 2009 | Triage | POC viral nasopharyngeal washing at nurse triage | Not reported |
| Keitel et al. 2019 | Add-on | POC CRP based on specific clinical assessment (age/temperature-corrected tachypnea and/or chest indrawing, and after trial of inhaled salbutamol) | Educational component incorporated into EPOCT decision algorithm. |
| Schot et al. 2018 | Add-on | POC CRP test was performed after clinical assessment by the treating GP. | Yes. GPs were not provided with strict decision rules based on CRP levels, but were given some interpretation guidance on CRP levels:  POC CRP levels should be interpreted in combination with symptoms and signs.  2. CRP levels <10mg/L make pneumonia less likely, but should not be used to exclude pneumonia when the GP finds the child ill, or when duration of symptoms is <6 hours.  3. CRP levels >100mg/L make pneumonia much more likely, however, such levels can also be  caused by viral infections.  4. Between 10mg/L and 100mg/L, the likelihood of pneumonia increases with increasing CRP levels. |
| **Acute sore throat** |  |  |  |
| Ayanruoh et al. 2009 | Replacement | RSTs were performed on all patients with pharyngitis during implementation phase. | Yes, in-service training session on correct sampling technique, performance, and interpretation of RST results by our microbiology consultant before the initiation of this study. |
| Bird et al. 2018 | Add-on | Treating clinicians could only request a POC, if McIsaac score was 3 or greater after clinical examination | POC training only |
| Malecki et al. 2017 | Add-on | Each patient received a POCT before a treatment plan was established by their clinician | Not reported. |
| Maltezou et al. 2008 | Add-on | POC after clinical examination in children with at least one clinical criterion of pharyngitis: fever (>38C), tonsillar exudate, tender enlarged anterior cervical lymph nodes and absence of cough. | POC training only |
| Meier et al. 1990 | Replacement | During implementation phase, technologist carried out a POC. Results were available during the same clinic visit at which a specimen was obtained. | Not reported |
| **HIV** |  |  |  |
| Bianchi et al. 2019 | Replacement | Caregiver presents with infant for HIV testing as part of routine care for infants exposed to HIV. | Initial training and ongoing technical support. |
| Jani et al. 2018 | Replacement | Eligible children post-randomisation HIV-exposed infants who presented at regular consultation visits | Yes, sites received brief clinical refresher training on best practices for the care cascade, early infant diagnosis algorithm, and paediatric antiretroviral therapy to ensure comparable quality of care. In both arms, nurses were encouraged to refer patients with HIV-positive results for antiretroviral therapy initiation on the same day of result availability, in line with Mozambique’s national guidelines and WHO recommendations |
| Mwenda et al. 2018 | Replacement | Routine clinical indication for POC test per national guidelines for infants exposed to HIV | Yes, accompanying clinical algorithms, and clinical training and practices were the same in both arms and across the period of the study. |
| **Insulin-dependent diabetes mellitus** |  |  |  |
| Agus et al. 2010 | Replacement | Patients randomized to intervention group had POC Hba1c before their clinic visit by a study technician. available to clinicians at the clinic visit | Not reported |

^a^ The intended roles were defined as ‘triage’, in which the new test is used at the start of the clinical pathway, ‘replacement’, in which the new test replaces an existing test, either as a faster equivalent test or to replace a non-point-of-care laboratory test, or ‘add-on’ in which the new test is performed at the end of a clinical pathway; Abbreviations: CHW Community Health Worker; POCT point-of-care test; POC point-of-care; RST rapid streptococcal test; RDT rapid diagnostic test; IMCI Integrated Management of Childhood Illness; CRP C-reactive protein; HIV Human Immunodeficiency Virus.

## **S3. Further information on patient outcomes in malarial studies**

In the Baiden et al. study, there was no statistically difference in mortality rates between POCT (1%; 15/1,527) versus usual care arms (1.4%; 21/1,519). (Baiden, Bruce et al. 2016) There was: one paediatric death in the Ansah et al. study(Ansah, Narh-Bana et al. 2010); two deaths in the intervention arm (2/963) and one in the control arm (1/2084) in the Zambian study(Yeboah-Antwi, Pilingana et al. 2010); and three children died in the Tanzanian study (two due to malaria, one in each arm).(Mubi, Janson et al. 2011)

## **S4. Timeliness of appropriate antimalarial treatment**

Based on two cluster RCTs (Mbonye, Magnussen et al. 2015, Ndyomugyenyi, Magnussen et al. 2016), the proportion of children with malaria receiving prompt and appropriate antimalarial treatment within 24 hours was significantly greater with rapid diagnostic tests compared to “usual care” whether that care was delivered by community health workers or registered drug shops (RR 2.72, 95% CI [1.15 to 6.43], n= 11,304)


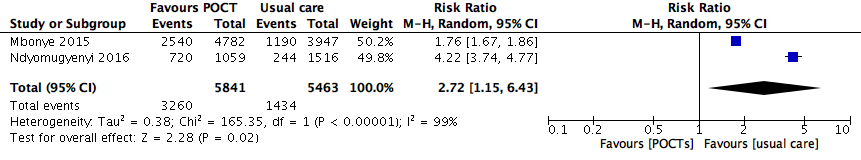


Fig 1. Prompt and appropriate antimalarial treatment within 24 hours. Forest plot of meta-analyses of randomised trials of children with malaria receiving prompt and appropriate antimalarial treatment within 24 hours comparing POCT vs usual care. Abbreviations: CI, confidence interval; POCT, point-of-care test; RCT, randomised controlled trial;

## **S5. Safety and antimalarial treatment**

In the context of safety and antimalarial treatment, the included studies provided five areas for scrutiny summarised in the Table below with a more detailed discussion below:

1. Between POCT and usual care
2. microscopy-positive patients and not prescribed antimalarials (false negatives);
3. microscopy-negative patients and prescribed antimalarials (false positives);
4. POCT-positive patients and not prescribed antimalarials (i.e. human error);
5. POCT-negative patients and prescribed antimalarials (i.e. distrust of result)
6. Data from POCT-arm only
7. POCT-positive patients and prescribed antibiotics (i.e. potential overprescribing);

Summary Table. Safety and antimalarial treatment

| **Outcome** | **Studies (n)** | **(Pooled) Effect estimate** |
| --- | --- | --- |
| Microscopy-positive children and not prescribed antimalarials | 3 RCTs (Ansah, Narh-Bana et al. 2010, Baiden, Bruce et al. 2016, Ndyomugyenyi, Magnussen et al. 2016) | RR 2.77,  95% CI [0.96 to 7.97], p=0.06, n= 7,328 |
| Microscopy-negative children and prescribed antimalarials | 3 RCTs (Ansah, Narh-Bana et al. 2015, Baiden, Bruce et al. 2016, Ndyomugyenyi, Magnussen et al. 2016) | RR 0.24,  95% CI [0.11 to 0.49], p<0.001, n= 9,041 |
| POCT-positive children and not prescribed antimalarials ^a^ | 1 Cluster RCT (Chandler, Webb et al. 2017) | RR 1.17,  95% CI [0.96 to 1.43], p=0.12, n=1,006) |
| POCT-negative children and prescribed antimalarials ^b^ | 2 Cluster RCTs (Mukanga, Tiono et al. 2012, Chandler, Webb et al. 2017) | RR 0.47,  95% CI [0.19 to 1.17], p=0.11, n= 1,309 |

^a^ In Chandler (2017) although POCTs were available in the ‘usual care’ arm, no training was provided nor adequate POCT supply^; b^ In Mukanga (2012), the authors estimated a similar proportion of RDT negative cases in the intervention and control cases to make this calculation

1. Microscopy-positive patients and not prescribed antimalarials

Without the use of specialised diagnostic equipment, antimalarials were more likely to be prescribed in ‘usual care’ for suspected malaria in children except for one study (Figure WW). (Baiden, Bruce et al. 2016) In the latter study, although the number of blood films that were positive was similar in both arms (50.3% versus 53.3%; p = 0.61), the number of children who were blood smear positive but did not receive antimalarial treatment was significantly higher in the control arm than in the POCT arm (12.5% versus 6.5%; p = 0.04). Given that the strongest predictor of a repeat episode of malaria is an index episode of parasitaemia, the authors conclude that there may have been higher false-negative diagnosis of parasitaemic children in the control arm that could have contributed to an increased risk of malaria in children in this group.


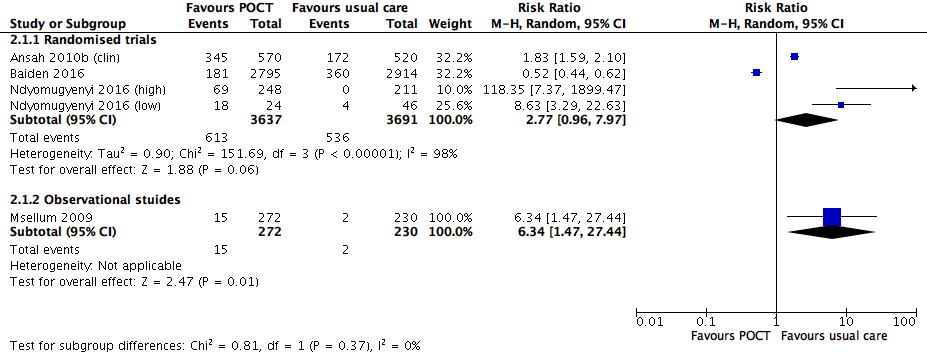


Fig 2. Forest plot of meta-analyses of randomised trials and observational studies of microscopy-positive patients not prescribed antimalarials comparing POCT vs usual care. Abbreviations: CI, confidence interval; POCT, point-of-care test; RCT, randomised controlled trial

1. Microscopy-negative patients and prescribed antimalarials

In contrast, the number of antimalarials used in malarial blood-film negative patients was significantly less with the use of POCTs compared to usual care (Fig 3). Based on three RCTs, the number of antimalarials used was 75% less with POCTs than in ‘usual care’.(Ansah, Narh-Bana et al. 2015, Baiden, Bruce et al. 2016, Ndyomugyenyi, Magnussen et al. 2016)


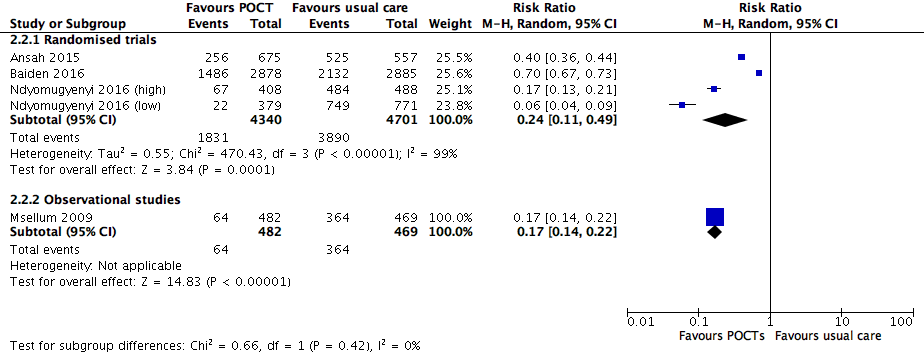


Fig 3. Forest plot of meta-analyses of randomised trials and observational studies of microscopy-negative patients prescribed antimalarials comparing POCT vs usual care. Abbreviations: CI, confidence interval; POCT, point-of-care test; RCT, randomised controlled trial

1. POCT-positive patients and not prescribed antimalarials

In the one study that evaluated POCT-positive patients not prescribed antimalarials in both intervention and control, there was no statistically significant difference (RR 1.17, 95% CI [0.96 to 1.43], n=1,006).(Chandler, Webb et al. 2017) In this study, although POCTs were available in the ‘usual care’ arm, no training was provided or assurance of adequate POCT supply.

1. POCT-negative patients and prescribed antimalarials

There was no statistical difference between POCT-negative children that were prescribed antimalarials for children receiving POCT or usual care (Fig 4). Readers are advised that In Mukanga (2012), the authors assumed a similar proportion of RDT negative cases in the intervention and control cases to estimate POCT-negative children who were prescribed antimalarials.


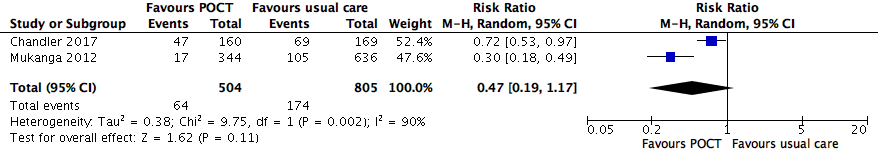


Fig 4. POCT-negative children and prescribed antimalarials. Forest plot of meta-analyses of randomised trials of the likelihood of antimalarial treatment in POCT-negative children comparing POCT vs usual care. Abbreviations: CI, confidence interval; POCT, point-of-care test; RCT, randomised controlled trial

1. POCT arm only: POCT-positive patients prescribed antibiotics

Likewise, considering available data of POCT arms only to assess the potential overprescribing of antibiotics in POCT-positive patients, 44% of malarial POCT-positive children received antibiotics (2,391/5,406, two RCTs and one observational study).(Baiden, Bruce et al. 2016, Chandler, Webb et al. 2017, Hopkins, Bruxvoort et al. 2017) This in comparison to 75% of POCT-negative children that received antibiotics (1,825/2,441).

## **S6. Additional information about point-of-care testing C-reactive protein in non-specific acute fever illness**

The median CRP (IQR mg/L), varied between studies: from ≤5 mg/L (≤5–6 mg/L) (Lemiengre, Verbakel et al. 2018); 7 mg/L (5–23 mg/L) (Verbakel, Lemiengre et al. 2016) without clinical risk score, 11 mg/L (5–30 mg/L) with clinical risk score; 18 mg/L (6–46 mg/L)(Nijman, Moll et al. 2015); 21 mg/L (0–154mg/L)(Van den Bruel, Jones et al. 2016) and not reported in one study.(Cohen, Lecuyer et al. 2008) One study employed pre-consultation screening with CRP of randomised children.(Rebnord, Sandvik et al. 2017) In this Norwegian study, the mean pretested CRP was significantly lower than when requested by the doctor (21 vs 34 mg/L, p=0.006). Here, the Norwegian guidelines recommend expectant management when CRP is below 50–100 mg/L at day 2 and later.

In the Althaus study, children were randomly assigned using a computer-based randomisation system at a ratio of 1:1:1 to either the control group or one of two CRP testing groups, which used thresholds of 20 mg/L (group A) or 40 mg/L CRP (group B) prior to clinical examination. Patients were then provided with a card specifying whether their CRP concentrations were high or low in relation to their intervention group and referred to the health-care provider.(Althaus, Greer et al. 2019)

## **S7. POCT-Hba1c and Diabetes Mellitus**

Table 2. POCT-Hba1c on clinical care in insulin-dependent children (n= 215).(Agus, Alexander et al. 2010)

| Baseline | 7.90 ± 1.24% (111) | 7.81 ± 1.13% (104) |
| --- | --- | --- |
|  | **Change in Hba1c Intervention Group (n)** | **Change in Hba1c Control Group (n)** |
| 3 months | −0.20 ± 0.66% (88) | −0.08 ± 0.72% (77) |
| 6 months | −0.03 ± 0.86% (81) | 0.02± 0.83% (72) |
| 9 months | 0.14 ± 0.98% (83) | 0.24 ± 1.07% (74) |
| 12 months | 0.14 ± 0.98% (77) | 0.24 ± 1.07% (64) |
| Post-study follow-up 15-24 months | 0.41 ± 1.03% (104) | 0.38 ± 1.15% (97) |

## **S8. Search strategy example of electronic database (Medline)**

|  | *Searches: 1946 to 29 January 2020* |
| --- | --- |
| 1 | Ambulatory Care/ |
| 2 | exp Ambulatory Care Facilities/ |
| 3 | general practice/ or family practice/ |
| 4 | general practitioners/ or physicians, family/ or physicians, primary care/ |
| 5 | Primary Health Care/ |
| 6 | Office Visits/ |
| 7 | exp Emergency Service, Hospital/ |
| 8 | Emergency Medical Services/ |
| 9 | (ambulatory adj3 (care or setting? or facilit* or ward? or department? or service?)).ti,ab. |
| 10 | ((general or family) adj2 (practi* or physician? or doctor?)).ti,ab. |
| 11 | (primary care or primary health care or primary healthcare).ti,ab. |
| 12 | (emergency adj3 (care or setting? or facilit* or ward? or department? or service?)).ti,ab. |
| 13 | (after hour? or afterhour? or "out of hour?" or ooh).ti,ab. |
| 14 | (clinic? or visit?).ti,ab. |
| 15 | ((health* or medical) adj2 (center? or centre?)).ti,ab. |
| 16 | community health services/ or exp community health nursing/ |
| 17 | Community Health Workers/ |
| 18 | (community adj2 (health or health care or service? or program*)).ti,ab. |
| 19 | (community adj2 (worker? or aide? or volunteer? or assistant? or visitor?)).ti,ab. |
| 20 | ((lay or volunteer) adj2 (health worker? or health aide? or health assistant?)).ti,ab. |
| 21 | ((health* or medical) adj2 (facility or facilities)).ti,ab. |
| 22 | 1 or 2 or 3 or 4 or 5 or 6 or 7 or 8 or 9 or 10 or 11 or 12 or 13 or 14 or 15 or 16 or 17 or 18 or 19 or 20 or 21 |
| 23 | Point-of-Care Systems/ |
| 24 | (("point of care" or POC) adj3 (test* or diagnos*)).ti,ab. |
| 25 | (("point of care" or POC) and (test* or diagnos*)).ti. |
| 26 | poct.ti,ab. |
| 27 | ((rapid or bedside or bed-side or "near patient") adj3 (test* or diagnos*)).ti,ab. |
| 28 | ((rapid or bedside or bed-side or "near patient") and (test* or diagnos*)).ti. |
| 29 | 23 or 24 or 25 or 26 or 27 or 28 |
| 30 | (istat or i-stat or afinion).ti,ab. |
| 31 | 29 or 30 |
| 32 | adolescent/ or exp child/ or exp infant/ |
| 33 | (child* or schoolchild* or preschooler* or pre-schooler* or girl* or boy* or infant* or baby or babies or teen* or adolescen*).ti,ab. |
| 34 | 32 or 33 |
| 35 | 22 and 31 and 34 |

References

Agus, M. S., J. L. Alexander and J. I. Wolfsdorf (2010). "Utility of immediate hemoglobin A1c in children with type I diabetes mellitus." Pediatr Diabetes **11**(7): 450-454.

Althaus, T., R. C. Greer, M. M. M. Swe, J. Cohen, N. N. Tun, J. Heaton, S. Nedsuwan, D. Intralawan, N. Sumpradit, S. Dittrich, Z. Doran, N. Waithira, H. M. Thu, H. Win, J. Thaipadungpanit, P. Srilohasin, M. Mukaka, P. W. Smit, E. N. Charoenboon, M. J. Haenssgen, T. Wangrangsimakul, S. Blacksell, D. Limmathurotsakul, N. Day, F. Smithuis and Y. Lubell (2019). "Effect of point-of-care C-reactive protein testing on antibiotic prescription in febrile patients attending primary care in Thailand and Myanmar: an open-label, randomised, controlled trial." Lancet Glob Health **7**(1): e119-e131.

Ansah, E. K., S. Narh-Bana, H. Affran-Bonful, C. Bart-Plange, B. Cundill, M. Gyapong and C. J. Whitty (2015). "The impact of providing rapid diagnostic malaria tests on fever management in the private retail sector in Ghana: a cluster randomized trial." Bmj **350**: h1019.

Ansah, E. K., S. Narh-Bana, M. Epokor, S. Akanpigbiam, A. A. Quartey, J. Gyapong and C. J. Whitty (2010). "Rapid testing for malaria in settings where microscopy is available and peripheral clinics where only presumptive treatment is available: a randomised controlled trial in Ghana." Bmj **340**: c930.

Baiden, F., J. Bruce, J. Webster, M. Tivura, R. Delmini, S. Amengo-Etego, S. Owusu-Agyei and D. Chandramohan (2016). "Effect of Test-Based versus Presumptive Treatment of Malaria in Under-Five Children in Rural Ghana--A Cluster-Randomised Trial." PLoS One **11**(4): e0152960.

Chandler, C. I., E. L. Webb, C. Maiteki-Sebuguzi, S. Nayiga, C. Nabirye, D. D. DiLiberto, E. Ssemmondo, G. Dorsey, M. R. Kamya and S. G. Staedke (2017). "The impact of an intervention to introduce malaria rapid diagnostic tests on fever case management in a high transmission setting in Uganda: A mixed-methods cluster-randomized trial (PRIME)." PLoS One **12**(3): e0170998.

Cohen, R., A. Lecuyer, C. Wollner, P. Deberdt, F. Thollot, V. Henriquet and F. de La Rocque (2008). "[Evaluation of impact of CRP rapid test in management of febrile children in ambulatory pediatric practice]." Arch Pediatr **15**(6): 1126-1132.

Diederichsen, H. Z., M. Skamling, A. Diederichsen, P. Grinsted, S. Antonsen, P. H. Petersen, A. P. Munck and J. Kragstrup (2000). "Randomised controlled trial of CRP rapid test as a guide to treatment of respiratory infections in general practice." Scand J Prim Health Care **18**(1): 39-43.

Do, N. T., N. T. Ta, N. T. Tran, H. M. Than, B. T. Vu, L. B. Hoang, H. R. van Doorn, D. T. Vu, J. W. Cals, A. Chandna, Y. Lubell, B. Nadjm, G. Thwaites, M. Wolbers, K. V. Nguyen and H. F. Wertheim (2016). "Point-of-care C-reactive protein testing to reduce inappropriate use of antibiotics for non-severe acute respiratory infections in Vietnamese primary health care: a randomised controlled trial." Lancet Glob Health **4**(9): e633-641.

Hopkins, H., K. J. Bruxvoort, M. E. Cairns, C. I. Chandler, B. Leurent, E. K. Ansah, F. Baiden, K. A. Baltzell, A. Bjorkman, H. E. Burchett, S. E. Clarke, D. D. DiLiberto, K. Elfving, C. Goodman, K. S. Hansen, S. P. Kachur, S. Lal, D. G. Lalloo, T. Leslie, P. Magnussen, L. M. Jefferies, A. Martensson, I. Mayan, A. K. Mbonye, M. I. Msellem, O. E. Onwujekwe, S. Owusu-Agyei, H. Reyburn, M. W. Rowland, D. Shakely, L. S. Vestergaard, J. Webster, V. L. Wiseman, S. Yeung, D. Schellenberg, S. G. Staedke and C. J. Whitty (2017). "Impact of introduction of rapid diagnostic tests for malaria on antibiotic prescribing: analysis of observational and randomised studies in public and private healthcare settings." Bmj **356**: j1054.

Jani, I. V., B. Meggi, O. Loquiha, O. Tobaiwa, C. Mudenyanga, A. Zitha, D. Mutsaka, N. Mabunda, A. Vubil, T. Bollinger, L. Vojnov and T. F. Peter (2018). "Effect of point-of-care early infant diagnosis on antiretroviral therapy initiation and retention of patients." Aids **32**(11): 1453-1463.

Lemiengre, M. B., J. Y. Verbakel, R. Colman, T. De Burghgraeve, F. Buntinx, B. Aertgeerts, F. De Baets and A. De Sutter (2018). "Reducing inappropriate antibiotic prescribing for children in primary care: a cluster randomised controlled trial of two interventions." Br J Gen Pract **68**(668): e204-e210.

Mbonye, A. K., P. Magnussen, S. Lal, K. S. Hansen, B. Cundill, C. Chandler and S. E. Clarke (2015). "A Cluster Randomised Trial Introducing Rapid Diagnostic Tests into Registered Drug Shops in Uganda: Impact on Appropriate Treatment of Malaria." PLoS One **10**(7): e0129545.

Mubi, M., A. Janson, M. Warsame, A. Martensson, K. Kallander, M. G. Petzold, B. Ngasala, G. Maganga, L. L. Gustafsson, A. Massele, G. Tomson, Z. Premji and A. Bjorkman (2011). "Malaria rapid testing by community health workers is effective and safe for targeting malaria treatment: randomised cross-over trial in Tanzania." PLoS One **6**(7): e19753.

Mukanga, D., A. B. Tiono, T. Anyorigiya, K. Kallander, A. T. Konate, A. R. Oduro, J. K. Tibenderana, L. Amenga-Etego, S. B. Sirima, S. Cousens, G. Barnish and F. Pagnoni (2012). "Integrated community case management of fever in children under five using rapid diagnostic tests and respiratory rate counting: a multi-country cluster randomized trial." Am J Trop Med Hyg **87**(5 Suppl): 21-29.

Mwenda, R., Y. Fong, T. Magombo, E. Saka, D. Midiani, C. Mwase, J. Kandulu, M. Wang, R. Thomas, J. Sherman and L. Vojnov (2018). "Significant Patient Impact Observed Upon Implementation of Point-of-Care Early Infant Diagnosis Technologies in an Observational Study in Malawi." Clin Infect Dis **67**(5): 701-707.

Ndyomugyenyi, R., P. Magnussen, S. Lal, K. Hansen and S. E. Clarke (2016). "Appropriate targeting of artemisinin-based combination therapy by community health workers using malaria rapid diagnostic tests: findings from randomized trials in two contrasting areas of high and low malaria transmission in south-western Uganda." Trop Med Int Health **21**(9): 1157-1170.

Nijman, R. G., H. A. Moll, Y. Vergouwe, Y. B. de Rijke and R. Oostenbrink (2015). "C-Reactive Protein Bedside Testing in Febrile Children Lowers Length of Stay at the Emergency Department." Pediatr Emerg Care **31**(9): 633-639.

Rebnord, I. K., H. Sandvik, A. B. Mjelle and S. Hunskaar (2017). "Factors predicting antibiotic prescription and referral to hospital for children with respiratory symptoms: secondary analysis of a randomised controlled study at out-of-hours services in primary care." BMJ Open **7**(1): e012992.

Van den Bruel, A., C. Jones, M. Thompson and D. Mant (2016). "C-reactive protein point-of-care testing in acutely ill children: a mixed methods study in primary care." Arch Dis Child **101**(4): 382-385.

Verbakel, J. Y., M. B. Lemiengre, T. De Burghgraeve, A. De Sutter, B. Aertgeerts, B. Shinkins, R. Perera, D. Mant, A. Van den Bruel and F. Buntinx (2016). "Should all acutely ill children in primary care be tested with point-of-care CRP: a cluster randomised trial." BMC Med **14**(1): 131.

Yeboah-Antwi, K., P. Pilingana, W. B. Macleod, K. Semrau, K. Siazeele, P. Kalesha, B. Hamainza, P. Seidenberg, A. Mazimba, L. Sabin, K. Kamholz, D. M. Thea and D. H. Hamer (2010). "Community case management of fever due to malaria and pneumonia in children under five in Zambia: a cluster randomized controlled trial." PLoS Med **7**(9): e1000340.
